# Aberrant left main coronary artery (ALMCA) arising from the right sinus of Valsalva with interarterial course

**DOI:** 10.11604/pamj.2022.42.308.36850

**Published:** 2022-08-24

**Authors:** Badr Boutakioute, Anass Chehboun

**Affiliations:** 1 Department of Radiology, Ar-Razi Hospital, Mohammed VI University Hospital Center, Marrakech, Morocco

**Keywords:** Coronary computed tomography, sinus of valsalva, interarterial course

## Image medicine

Here, we report a case of an 80-year-old man with a history of hypertension and recurrent episodes of syncope and chest pain. First, he underwent a coronarography with revascularization of the right coronary artery and stent placement. The exploration of the left main coronary artery (LMCA) was not performed because of catheterization difficulties. A coroscanner was performed for suspicion of an anomalous of the left coronary artery. The coroscanner showed an uncommon case of left main coronary artery (LMCA) arising from the right sinus of Valsalva. The proximal part of the interventricular artery has an intra-arterial course between the aorta and the pulmonary outflow tract, with narrowing of this artery at this level (2.2 mm in diameter). Left main coronary artery (LMCA) or left anterior descending coronary artery (LAD) arising from the right sinus of Valsalva or right coronary artery (RCA) is referred to as an anomalous aortic origin of a coronary artery (AAOCA). The subsequent course is mostly between the aorta and the pulmonary artery on its way to the left ventricle. Different theories have been postulated as a cause of sudden death in these patients. The most accepted theory is the higher incidence of occlusion of the osmium secondary to a more slit-like orifice and occlusion during physical activity due to compression between the major arteries.

**Figure 1:**
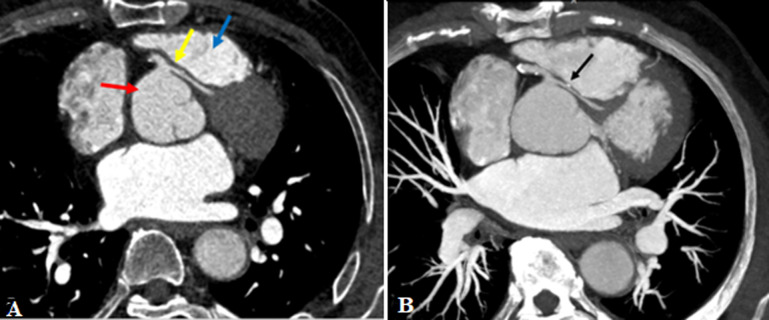
computed tomography coronary scan; A) axial reconstruction of computed tomography (CT) coronary angiogram showing the anomalous of the left main coronary artery, with a course (yellow arrow) between the aorta (red arrow) and the pulmonary outflow track (blue arrow); B) maximum intensity projection reconstruction (MIP) showing the narrowing of the left main coronary artery (LMCA) mesuring 2.2 mm at this level (black arrow)

